# Diagnostic value of the motor band sign in amyotrophic lateral sclerosis: a 7T magnetic resonance imaging study

**DOI:** 10.1186/s40035-025-00491-8

**Published:** 2025-06-18

**Authors:** Xunyan Huang, Zhe Zhang, Lin Chen, Shuo Yang, Xinyao Liu, Jingfeng Bi, Zaiqiang Zhang, Yongjun Wang, Ning Wei, Wanlin Zhu, Na Chen, Lin Hua, Yuan Li, Yilong Wang, Jing Jing, Hua Pan

**Affiliations:** 1 Department of Neurology, Beijing Tiantan Hospital, Capital Medical University, No. 119 S Fourth Ring West Rd, Fengtai District, Beijing, 100070 China; 2 China National Clinical Research Center for Neurological Diseases, Beijing, 100070 China; 3 Tiantan Neuroimaging Center of Excellence, Beijing Tiantan Hospital, Capital Medical University, Beijing, 100070 China; 4 School of Biomedical Engineering, Capital Medical University, Beijing, 100069 China; 5 Siemens Healthineers, MR Research Collaboration Team, Beijing, 100102 China

Amyotrophic lateral sclerosis (ALS) is a fatal neurodegenerative disease affecting both upper and lower motor neurons, with a median survival of 3–5 years [1]. The key challenge in diagnosis lies in the early detection of upper motor neuron (UMN) impairment, which mainly depends on clinical examination but can be obscured by severe lower motor neuron (LMN) impairment [2]. Consequently, searching for alternative UMN impairment markers has become a critical focus of ALS research.

Recent magnetic resonance imaging (MRI) studies have indicated a band-shaped low signal intensity along the primary motor cortex (M1), termed the motor band sign (MBS) [3]. MBS has emerged as an imaging marker for UMN impairment in ALS [2, 4, 5]. Researchers believe this hypointensity results from ferritin accumulation within activated microglia in M1 [6]. Susceptibility-weighted imaging (SWI) has demonstrated increased sensitivity in detecting subtle, uniformly distributed iron deposits, becoming the current mainstream modality for identifying MBS. However, there is currently a lack of reports of MBS in 7T SWI.

To evaluate the diagnostic efficacy of the MBS by 7T SWI in identifying UMN impairment, we prospectively recruited an ALS cohort at Beijing Tiantan Hospital between October 2021 and March 2023 (Fig. S1). All participants underwent standardized physical examinations, electromyography, and 7T MRI within a week. Quantitative assessment of MBS was performed using the motor band hypointensity ratio (MBHR) (Fig. 1a), with details provided in Additional file 2 (The MBHR measurement protocol).

**Fig. 1:**
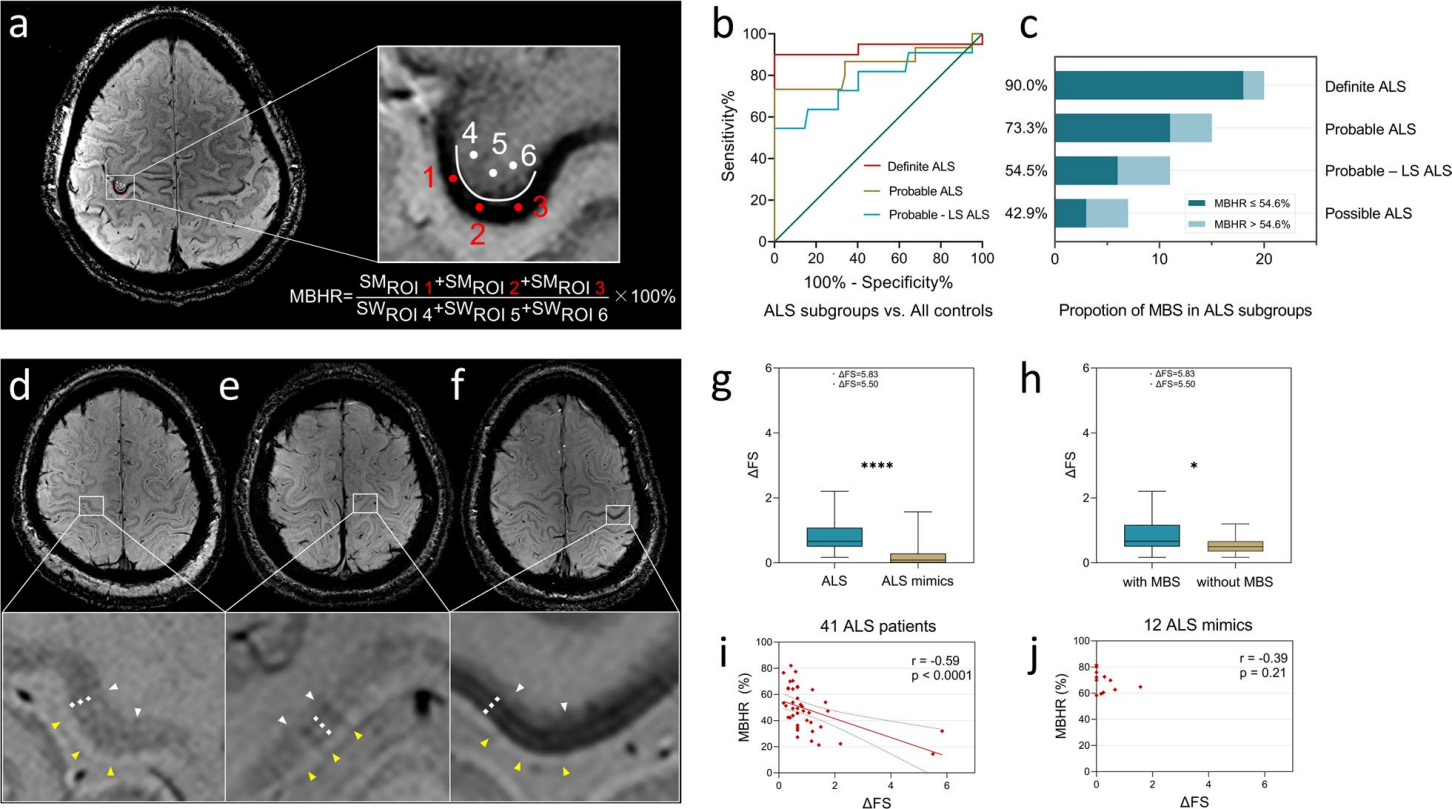
**a** ROI selection, MBHR calculation formula, and enlarged MBS schematic in 7T SWI from an ALS patient. **b** ROC analysis for differentiation of ALS subgroups from all controls. **c** Positive rates of MBS across ALS subgroups. **d**–**f** Magnified M1 regions in 7T SWI of a healthy control (left), an HSP patient (middle), and an ALS patient (right). **g**, **h** ΔFS comparisons between ALS and ALS mimics (*P* < 0.0001) and between ALS with MBS and without MBS (*P* = 0.015). Two outlier ALS patients with rapid progression were excluded. **i**, **j** Correlation analyses of MBHR with ΔFS in ALS patients (*r* = − 0.51, *P* = 0.0006) and ALS mimics (*r* = − 0.33, *P* = 0.30). ROIs, regions of interest; SWI, susceptibility-weighted imaging; ALS, amyotrophic lateral sclerosis; ROC, receiver operating characteristic; MBS, motor band sign; M1, primary motor cortex; HSP, hereditary spastic paraplegia; ΔFS, disease progression rate; MBHR, motor band hypointensity ratio; probable-LS ALS, probable laboratory-supported ALS

The ALS cohort comprised 20 clinically definite ALS, 15 clinically probable ALS, 11 clinically probable laboratory-supported ALS, and 7 clinically possible ALS. Age- and gender-matched controls (*n* = 62) included 50 healthy controls (HCs) and 12 ALS mimic cases: 6 patients with hereditary spastic paraplegia and 6 LMN syndrome patients. Demographic and clinical characteristics are detailed in Table S1.

The results of group differences (Kruskal–Wallis H test, details in Additional file 2: Statistical analysis) revealed significant MBHR variations among ALS patients, ALS mimics, and HCs (*P* < 0.0001; Fig. S2a). Specifically, ALS patients exhibited lower MBHR compared to both ALS mimics (*P* < 0.0001) and HCs (*P* < 0.0001). Significant MBHR differences were also observed among ALS subgroups, ALS mimics, and HCs (*P* < 0.0001; Fig. S2b). Additionally, no correlation was observed between MBHR and age in the HC group (*r* = − 0.08, *P* = 0.60; Fig. S3). Notably, we verified a significant association between MBHR and UMN impairment in ALS (Fig. S4), confirming the potential of MBS as a promising neuroimaging biomarker for ALS UMN impairment.

Moreover, receiver operating characteristic (ROC) analysis demonstrated the diagnostic performance of the MBS in differentiating clinically definite ALS patients from all controls (red line in Fig. 1b). MBHR cutoff of 54.6% yielded an area under the curve (AUC) of 0.930 (95% confidence interval [CI] 0.828–1.000), with 90.0% sensitivity and 100% specificity. Application of this threshold revealed differential MBS prevalence across ALS diagnostic subgroups: 90.0% in clinically definite ALS, 73.3% in clinically probable ALS, 54.5% in clinically probable laboratory-supported ALS, and 42.9% in clinically possible ALS (Fig. 1c). The demographics and clinical characteristics of ALS subgroups with MBHR ≤ 54.6% are provided in Table S2. ROC analysis of the utility of MBS for other ALS subgroups demonstrated AUC of 0.847 (95% CI 0.699–0.994) with 73.3% sensitivity and 98.4% specificity for clinically probable ALS patients versus all controls (brown line, Fig. 1b), as well as AUC of 0.777 (95% CI 0.591–0.964) with 54.5% sensitivity and 98.4% specificity for clinically probable laboratory**-**supported ALS patients versus controls (blue line, Fig. 1b).

The clinically definite/probable ALS patients exhibited significantly higher MGH upper motor neuron scales (*P* = 0.028) and lower revised amyotrophic lateral sclerosis functional rating scale (ALSFRS-R) scores (*P* = 0.027) compared to clinically probable laboratory-supported/possible ALS patients (Table S3). These functional differences may underlie the observed reduction of MBS prevalence in the latter groups.

Additionally, comparative analysis revealed significantly faster disease progression in ALS patients versus ALS mimics (*P* < 0.0001; Fig. 1g). Among ALS patients, those exhibiting the MBS demonstrated accelerated progression compared to MBS-negative cases (*P* = 0.015; Fig. 1h). Furthermore, a strong negative correlation between MBHR and disease progression rate (ΔFS) was observed in ALS patients (*r* = − 0.51, *P* = 0.0006; Fig. 1i), whereas no significant association was observed in ALS mimics (*r* = − 0.33, *P* = 0.30; Fig. 1j). The significant association remained after removal of two outliers with rapid progression in the ALS group (Fig. S5).

Conventional approaches for MBS evaluation face challenges: subjective variability in visual assessments and technical limitations of quantitative methods (more details in Additional file 2: The MBHR measurement protocol). To overcome these limitations, our study used the adjacent subcortical white matter region as the reference standard, with two primary goals: enhancing clinical practicality through simplified protocol implementation and mitigating artifacts caused by 7T magnetic field inhomogeneities. This optimized SWI protocol demonstrated high interobserver consistency (Additional file 2: Intergroup consistency).

Our results revealed a progressive decline in diagnostic sensitivity across ALS subgroups, paralleling reductions in diagnostic accuracy. Clinically probable laboratory-supported/possible ALS patients demonstrated significantly higher ALSFRS-R scores compared to clinically definite/probable ALS patients, suggesting earlier disease stages. The clinically probable laboratory-supported/possible ALS subgroups also exhibited milder UMN impairment, further indicating the MBS as a biomarker correlating with advanced disease burden and UMN degeneration severity. Although MBS detection rate was lower in these patients, a positive MBS can greatly boost diagnostic confidence. Integrating MBS as an additional UMN marker may accelerate diagnosis in ambiguous cases. For early-stage patients testing negative for MBS, follow-up MRI scans during disease progression could improve detection.

In a subset of eight ALS patients with available clinical 3T MRI data, 7T SWI demonstrated superior MBS detection rates (7/8 vs. 4/8 with 3T SWI) and provided enhanced visualization of lesion internal architecture (Figs. S6 and S7). The study revealed that some ALS patients demonstrated an explicit stratified pattern on 7T SWI. We observed three layers (white rhomboids) between the hyperintense superficial grey matter layers (yellow arrowheads) and the grey-white matter junction (white arrowheads) in M1 in healthy controls as well as in ALS mimics (Fig. 1d, e). In ALS patients, the signal intensity of the superficial and the deep layers in these three layers decreased, resulting in an Oreo-fashion (dark-bright-dark)-layered MBS (Fig. 1f). Detailed MBS images from all ALS patients are provided in Figs. S8–S10.

Recent functional MRI studies have confirmed laminar-specific cortical activation patterns in humans [7], consistent with histological evidence of ferritin-rich microglia predominantly localized in the middle and deep layers of the M1 [6]. Our findings suggest that the observed Oreo-fashion-layered MBS may reflect the cytoarchitecture organization of M1. However, Northall et al. reported predominant iron deposition in M1 layer VI (deepest cortical layer) in ALS patients [8]. This discrepancy may be explained by the lack of SWI data and the heterogeneous grouping of MBS-positive/negative cohorts. Therefore, future research integrating submillimeter ultra-high-field MRI, iron-sensitive imaging, and disease pathology is necessary for further understanding the layer-specific pathological features of ALS.

The identification of biomarkers for disease progression is critical for managing and treating ALS. Our findings support accelerated progression in ALS patients with MBS and a strong inverse correlation between MBHR and ΔFS. Thus, we propose that MBS strongly correlates with disease progression, warranting more consideration in clinical practice.

There are several limitations in this study. First, the small sample sizes of ALS patients and disease controls limit the generalization of our results. A large proportion of patients were lost to follow-up, which may reduce the statistical power, although the statistical and demographic data were comparable between follow-up and loss of follow-up populations (Table S4). Second, this study lacked cognitive data and follow-up imaging data; these limitations should be addressed in future research. Third, scanners from different vendors, as well as differences in field strengths and imaging parameters (e.g., voxel size, slice thickness) may affect the image signal values on which MBHR measurement is based and consequently influence the results. Thus, cross-validation studies at different centers using different acquisition protocols in larger cohorts are required to confirm and improve the diagnostic potency and robustness of this evaluation methodology.

In conclusion, MBS as quantified by MBHR (≤ 54.6%) on 7T SWI, shows strong potential for detecting UMN involvement in ALS, and correlates significantly with disease severity and progression. Future multi-center, longitudinal investigations are necessary to validate these findings, refine optimal MBHR thresholds, and elucidate the longitudinal trajectory of MBS throughout disease evolution.

## Supplementary Information

Additional file 1. **Table S1** Demographic data and clinical data of ALS, ALS mimics and HCs. **Table S2** Demographic and clinical data between ALS subgroups whose MBHR ≤ 54.6%. **Table S3** The comparison of MGH UMNSs and ALSFRS-R1 between ALS subgroups. **Table S4** The comparison of demographic and clinical data between patients who completed follow-up and those excluded. **Fig. S1** Flow chart of this study. **Fig. S2** Comparison of MBHR among ALS, ALS mimics, and HCs. **Fig. S3** Correlation analysis between MBHR and age in healthy controls. **Fig. S4** Correlation analyses between MBHR and MGH UMNSs in ALS patients. **Fig. S5** Correlation analysis between MBHR and ΔFS in ALS patients, 2 outlier patients with rapid progression were excluded. **Fig. S6** 7T and 3T SWI images of 4 ALS patients. **Fig. S7** 7T and 3T SWI images of 4 other ALS patients. **Fig. S8** Motor band signs in 7T SWI of clinically definite ALS patients a–r. **Fig. S9** Motor band signs in 7T SWI of clinically probable ALS patients a–k. **Fig.** **S10** Motor band signs in 7T SWI of clinically probable laboratory–supported ALS patients a–f; and clinically possible ALS patients g–i.

Additional file 2. Methods.

## Data Availability

These data that support the findings of this study are available from the corresponding author upon reasonable request.

## Abbreviations

ALS: Amyotrophic lateral sclerosis
ALSFRS-R: Revised amyotrophic lateral sclerosis functional rating scale
LMN: Lower motor neuron
M1: Primary motor cortex
MBHR: Motor band hypointensity ratio
MBS: Motor band sign
MRI: Magnetic resonance imaging
ROC: Receiver operating characteristic
SWI: Susceptibility-weighted imaging
UMN: Upper motor neuron
ΔFS: Disease progression rate
