# Supplementary material for: Diagnostic value of the motor band sign in amyotrophic lateral sclerosis: a 7T magnetic resonance imaging study
Source: Transl Neurodegener. 2025 Jun 18;14:30. doi: 10.1186/s40035-025-00491-8 (PMC12175456; doi:10.1186/s40035-025-00491-8)
Supplement: Supplementary file 1 — Additional file 1. Table S1 Demographic data and clinical data of ALS, ALS mimics and HCs. Table S2 Demographic and clinical data between ALS subgroups whose MBHR ≤ 54.6%. Table S3 The comparison of MGH UMNSs and ALSFRS-R1 between ALS subgroups. Table S4 The comparison of demographic and clinical data between patients who completed follow-up and those excluded. Fig. S1 Flow chart of this study. Fig. S2 Comparison of MBHR among ALS, ALS mimics, and HCs. Fig. S3 Correlation analysis between MBHR and age in healthy controls. Fig. S4 Correlation analyses between MBHR and MGH UMNSs in ALS patients. Fig. S5 Correlation analysis between MBHR and ΔFS in ALS patients, 2 outlier patients with rapid progression were excluded. Fig. S6 7T and 3T SWI images of 4 ALS patients. Fig. S7 7T and 3T SWI images of 4 other ALS patients. Fig. S8 Motor band signs in 7T SWI of clinically definite ALS patients a–r. Fig. S9 Motor band signs in 7T SWI of clinically probable ALS patients a–k. Fig. S10 Motor band signs in 7T SWI of clinically probable laboratory–supported ALS patients a–f; and clinically possible ALS patients g–i. [file 40035_2025_491_MOESM1_ESM.docx]

| **Demographic and clinical variables** | **ALS (N = 53)** | **ALS mimics (N = 12)** | | **HCs (N = 50)** | ***P* value** |
| --- | --- | --- | --- | --- | --- |
| Age, mean (SD), y | 54.87 (12.41) | 47.17 (12.61) | | 52.12 (6.78) | 0.131^a^ |
| Sex, no. (%) | | | | | |
| Male | 28 (52.83) | 7 (58.33) | | 22 (44.00) | 0.545^b^ |
| Female | 25 (47.17) | 5 (41.67) | | 28 (56.00) |  |
| Diagnosis by Revised EEC, no. (%) | | | | | |
| Clinically definite ALS | 20 (37.74) | NA | | NA | NA |
| Clinically probable ALS | 15 (28.30) |  |  |  |  |
| Clinically probable – LS ALS  Clinically possible ALS | 11 (20.75)  7 (13.21) |  |  |  |  |
| MGH UMNSs, mean (SD) | 27.04 (8.37) | HSP | LMN Syndrome* | NA | **0.003**^a^ |
|  |  | 30.50 (2.81) | 15.17 (3.37) |  |  |
| Disease duration, median (IQR), mo. | 13.00 (7.00-20.00) | 24.00 (17.25-81.00) | | NA | **0.001**^c^ |
| Interval between baseline and follow-up**, median (IQR), mo. | 6.00 (5.50-8.00) | 6.00 (6.00-7.00) | | NA | 0.393^c^ |
| ALSFRS-R1, median (IQR) | 42.00 (39.00-44.00) | 43.00 (42.00-44.00) | | NA | 0.174^c^ |
| ALSFRS-R2^d^, median (IQR) | 37.00 (31.00-40.00) | 42.00 (39.25-43.00) | | NA | **0.003**^c^ |
| ΔFS^d^, median (IQR) | 0.67 (0.50-1.16) | 0.08 (0.00-0.45) | | NA | **< 0.0001**^c^ |

**Table S1 Demographic data and clinical data of ALS, ALS mimics and HCs**

Statistical significance was defined as *P* < 0.05. Abbreviations: ALS, amyotrophic lateral sclerosis; HCs, healthy controls; Clinically probable – LS ALS, clinically probable laboratory–supported ALS; Revised EEC, revised El Escorial criteria; MGH UMNSs, MGH upper motor neuron scales; HSP, hereditary spastic paraplegia; LMN Syndrome, lower motor neuron syndrome; ALSFRS-R1, revised amyotrophic lateral sclerosis functional rating scale at baseline; ALSFRS-R2, revised amyotrophic lateral sclerosis functional rating scale at follow-up; ΔFS, disease progression rate, SD, standard deviation; IQR, interquartile range; NA, not available.

^a^ Kruskal-Wallis H test

^b^ Chi-square test

^c^ Mann-Whitney U test

^d^ Forty-one ALS patients and twelve ALS mimics were followed up.

* Include 4 chronic inflammatory demyelinating polyradiculoneuropathy (CIDP) patients, 2 multifocal motor neuropathy (MMN) patients.

** The mean follow-up interval was 6.61 months for ALS patients and 7.17 months for ALS mimics.

**Table S2 Demographic and clinical data between ALS subgroups whose MBHR ≤ 54.6%**

| **Clinical variables** | **Clinically definite ALS (N = 18)** | **Clinically probable ALS (N = 11)** | **Clinically probable – LS ALS (N = 6)** | **Clinically possible ALS (N =3)** |
| --- | --- | --- | --- | --- |
| Age, mean (SD), y | 51.78 (14.72) | 55.36 (13.08) | 50.33 (7.92) | 65.67 (9.50) |
| Sex, no. (%) |  |  |  |  |
| Male | 6 (33.33) | 7 (63.64) | 1 (16.67) | 2 (66.67) |
| Female | 12 (66.67) | 4 (36.36) | 5 (83.33) | 1 (33.33) |
| MGH UMNSs, mean (SD) | 33.00 (5.74) | 27.73 (8.26) | 27.50 (6.44) | 23.00 (11.53) |
| Disease duration, median (IQR), mo. | 15.00 (7.00-21.00) | 12.00 (6.00-13.00) | 11.50 (6.75-14.75) | 38.00 (7.00-NA) |
| ALSFRS-R1, median (IQR) | 39.00 (35.75-42.50) | 41.00 (37.00-44.00) | 44.00 (40.25-46.00) | 42.00 (42.00-NA) |
| ALSFRS-R2^d^, median (IQR) | 35.00 (24.00-41.50) | 34.00 (28.50-38.25) | 38.50 (31.00-39.25) | 43.00 (43.00-43.00) |
| ΔFS^d^, median (IQR) | 1.00 (0.50-1.93) | 0.81 (0.67-1.45) | 0.67 (0.53-0.98) | 0.50 (0.50-0.50) |
| MBHR, mean (SD) | 35.15 (13.57) | 40.16 (9.79) | 41.15 (6.65) | 52.20 (2.29) |

Abbreviations: ALS, amyotrophic lateral sclerosis; MGH UMNSs, MGH upper motor neuron scales; ALSFRS-R1, revised amyotrophic lateral sclerosis functional rating scale at baseline; ALSFRS-R2, revised amyotrophic lateral sclerosis functional rating scale at follow-up; SD, standard deviation; IQR, interquartile range; MBHR, motor band hypointensity ratio; NA, not available.

**Table S3 The comparison of MGH UMNSs and ALSFRS-R1 between ALS subgroups**

| **Clinical variables** | **Clinically definite/probable ALS (N = 35)** | **Clinically probable – LS/ possible ALS (N = 18)** | ***P* value** |
| --- | --- | --- | --- |
| MGH UMNSs, mean (SD) | 30.94 (6.08) | 22.83 (8.54) | **0.028**^a^ |
| ALSFRS-R1, median (IQR) | 39.00 (35.50 - 43.50) | 43.00 (42.00 - 44.00) | **0.027**^b^ |

Statistical significance was defined as *P* < 0.05. Abbreviations: Clinically probable – LS ALS, clinically probable laboratory–supported ALS; MGH UMNSs, MGH upper motor neuron scales; ALSFRS-R1, revised amyotrophic lateral sclerosis functional rating scale at baseline; SD, standard deviation; IQR, interquartile range.

^a^ Independent-Sample T Test

^b^ Mann-Whitney U test

**Table S4 Demographic and clinical data between patients who completed follow-up and those excluded**

| **Clinical variables** | **ALS patients under follow-up**  **(N = 41)** | **ALS patients lost to follow-up**  **(N = 12)** | ***P* value** |
| --- | --- | --- | --- |
| Age, mean (SD), y | 54.37 (12.20) | 56.58 (13.54) | 0.614^a^ |
| Sex, no. (%) |  |  |  |
| Male | 21 (51.20) | 7 (58.30) | 0.664^b^ |
| Female | 20 (48.80) | 5 (41.70) |  |
| MGH UMNSs, mean (SD) | 25.83 (7.90) | 31.17 (8.94) | 0.936^a^ |
| Disease duration, median (IQR), mo. | 14 (7.00-24.00) | 15 (6.25-22.50) | 0.757^c^ |
| ALSFRS-R1, median (IQR) | 43.0 (39.50-44.00) | 42.0 (39.25-44.25) | 0.856^c^ |
| MBHR, mean (SD) | 48.55 (16.36) | 40.92 (16.71) | 0.905^a^ |

Statistical significance was defined as *P* < 0.05. Abbreviations: ALS, amyotrophic lateral sclerosis; MGH UMNSs, MGH upper motor neuron scales; ALSFRS-R1, revised amyotrophic lateral sclerosis functional rating scale at baseline; MBHR, motor band hypointensity ratio; SD, standard deviation; IQR, interquartile range.

^a^ Independent-sample T test

^b^ Chi-square test

^c^ Mann-Whitney U test


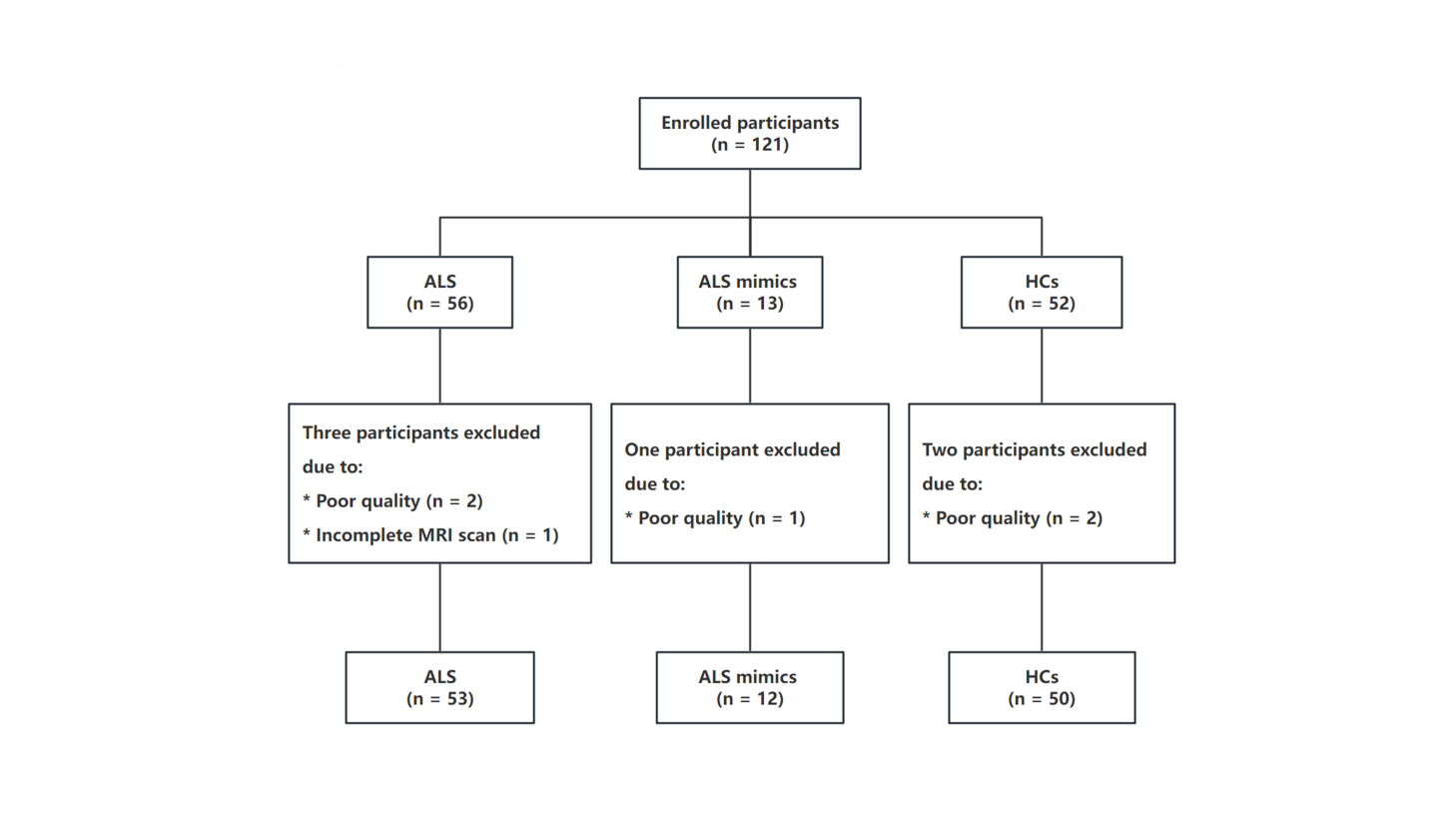


**Fig. S1 Flowchart of this study**

Abbreviations: ALS, amyotrophic lateral sclerosis; HCs, healthy controls; MRI, magnetic resonance imaging.

**
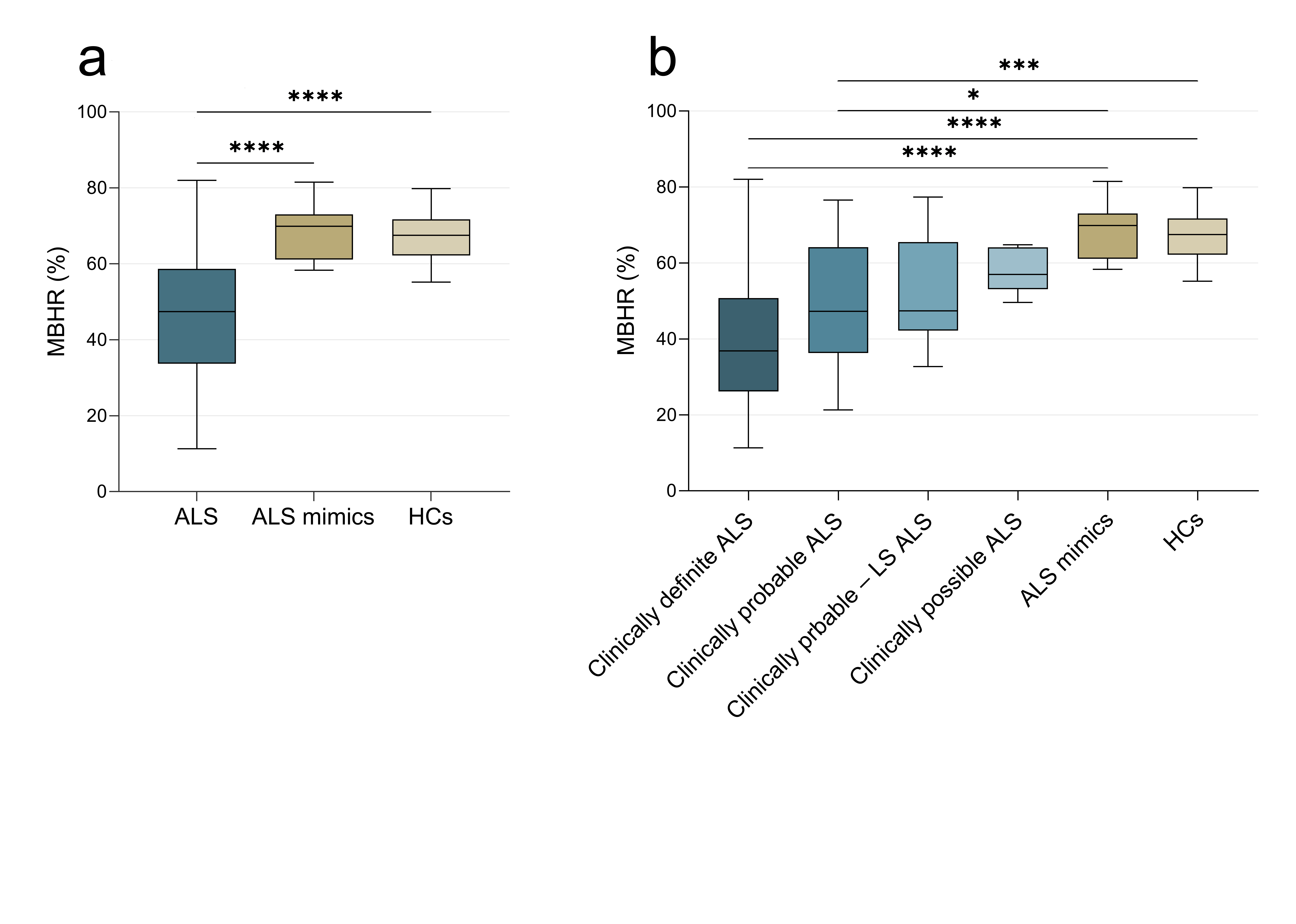
**

**Fig. S2 Comparison of MBHR among ALS, ALS mimics, and HCs**

(**a**) MBHR was significantly lower in ALS patients compared to both ALS mimics (*P* < 0.0001) and HCs (*P* < 0.0001). (**b**) Subgroup analysis revealed that MBHR reductions were particularly prominent in clinically definite ALS (vs. ALS mimics: *P* < 0.0001; vs. HCs: *P* < 0.0001) and clinically probable ALS (vs. ALS mimics: *P* = 0.01; vs. HCs: *P* = 0.0006) subgroups. However, no statistically significant differences were observed in clinically probable – LS ALS (vs. ALS mimics: *P* = 0.22; vs. HCs: *P* = 0.07) or clinically possible ALS (vs. ALS mimics: *P* = 0.45; vs. HCs: *P* = 0.28) subgroups. Abbreviations: MBHR, motor band hypointensity ratio; ALS, amyotrophic lateral sclerosis; HCs, healthy controls; clinically probable – LS ALS, clinically probable laboratory–supported ALS.

**P* < 0.05 ***P* < 0.01 ****P* < 0.001 *****P* < 0.0001.


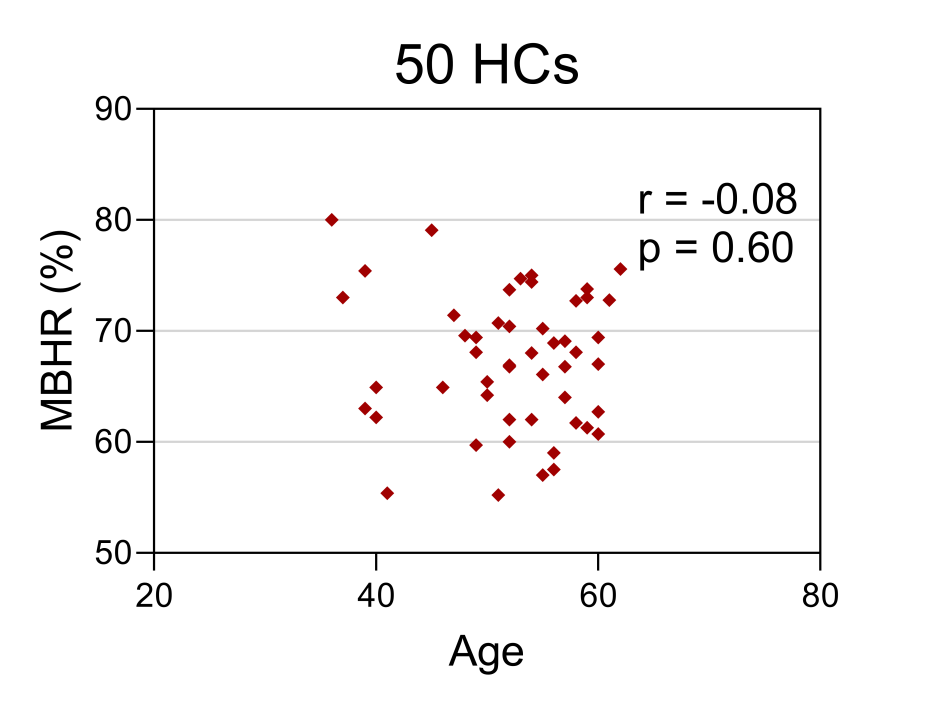


**Fig. S3 Correlation analyses between MBHR and age in HCs**

No correlation was observed between MBHR and age in 50 HCs (*r*= −0.08, *P*= 0.60). Abbreviations: MBHR, motor band hypointensity ratio; HCs, healthy controls.


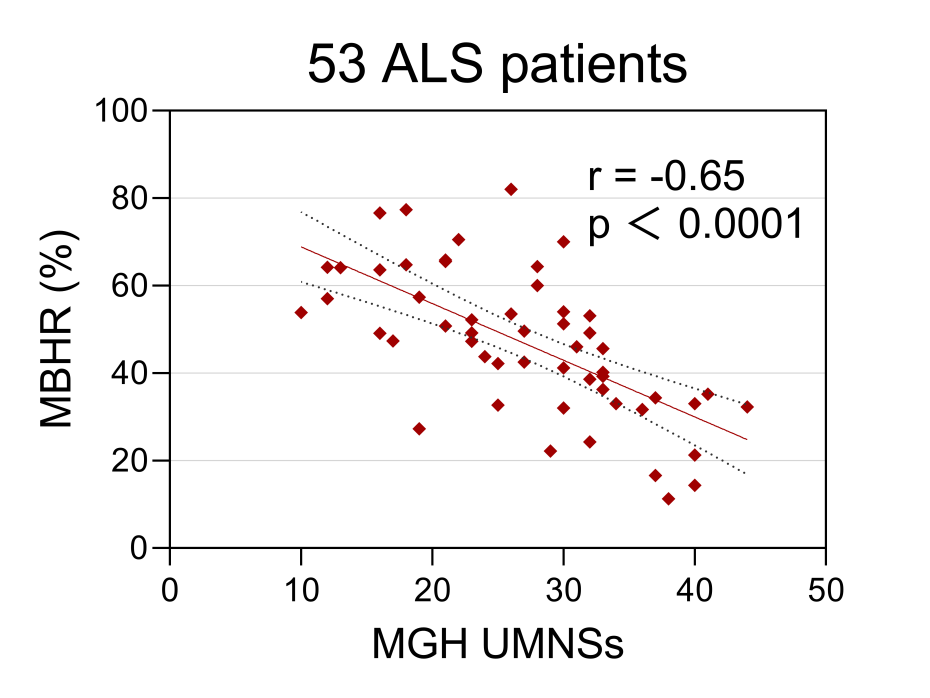


**Fig. S4 Correlation analysis between MBHR and MGH UMNSs in ALS patients**

A negative correlation was observed between MBHR and MGH UMNSs in 53 ALS patients (*r* = −0.65, *P* < 0.0001). Abbreviations: MBHR, motor band hypointensity ratio; MGH UMNSs, MGH upper motor neuron scales; ALS, amyotrophic lateral sclerosis.


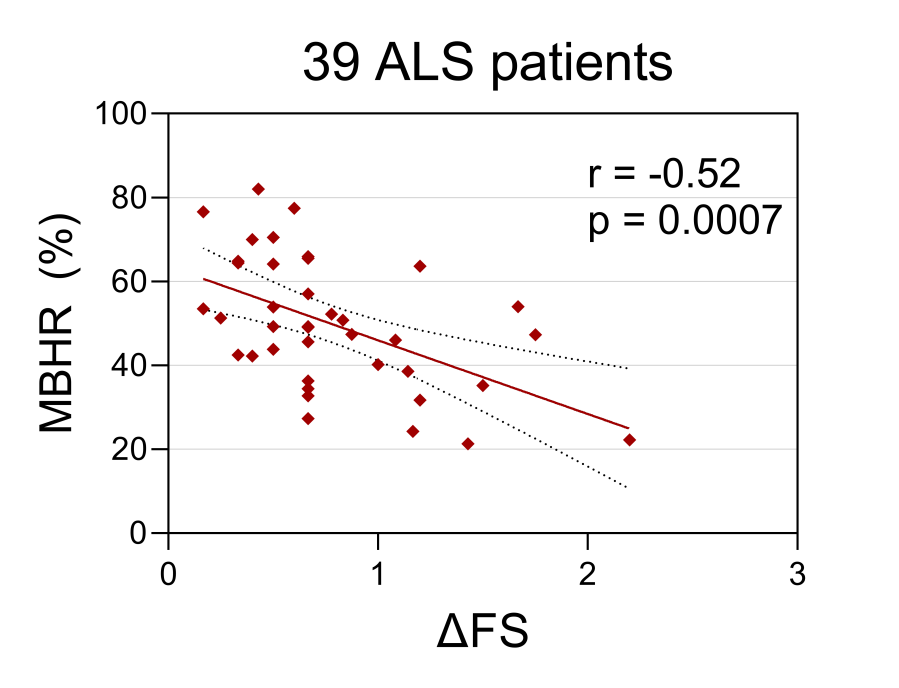


**Fig. S5 Correlation analysis between MBHR and ΔFS in ALS patients, 2 outlier patients with rapid progression were excluded**

A strong negative correlation between MBHR and ΔFS was observed in 39 ALS patients (*r* = −0.52, *P* = 0.0007). Abbreviations: ALS, amyotrophic lateral sclerosis; MGH UMNSs, MGH upper motor neuron scales; MBHR, motor band hypointensity ratio.


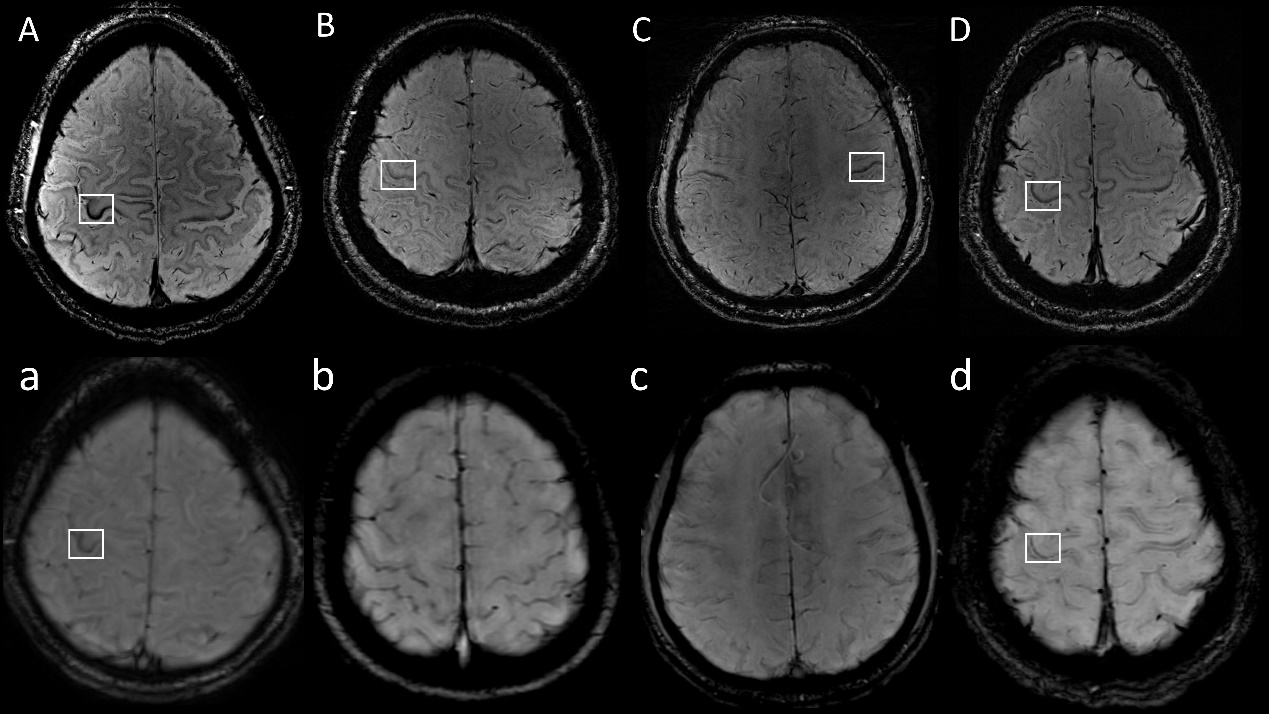
**Fig. S6 7T and 3T SWI images of 4 ALS patients**

Upper panels A-D display 7T SWI images from the four ALS patients, with corresponding 3T SWI images in lower panels a-d. White boxes indicate MBS confirmation using the MBHR threshold of 54.6%. The MBS was identified in all of the shown 7T cases and two of the 3T cases.


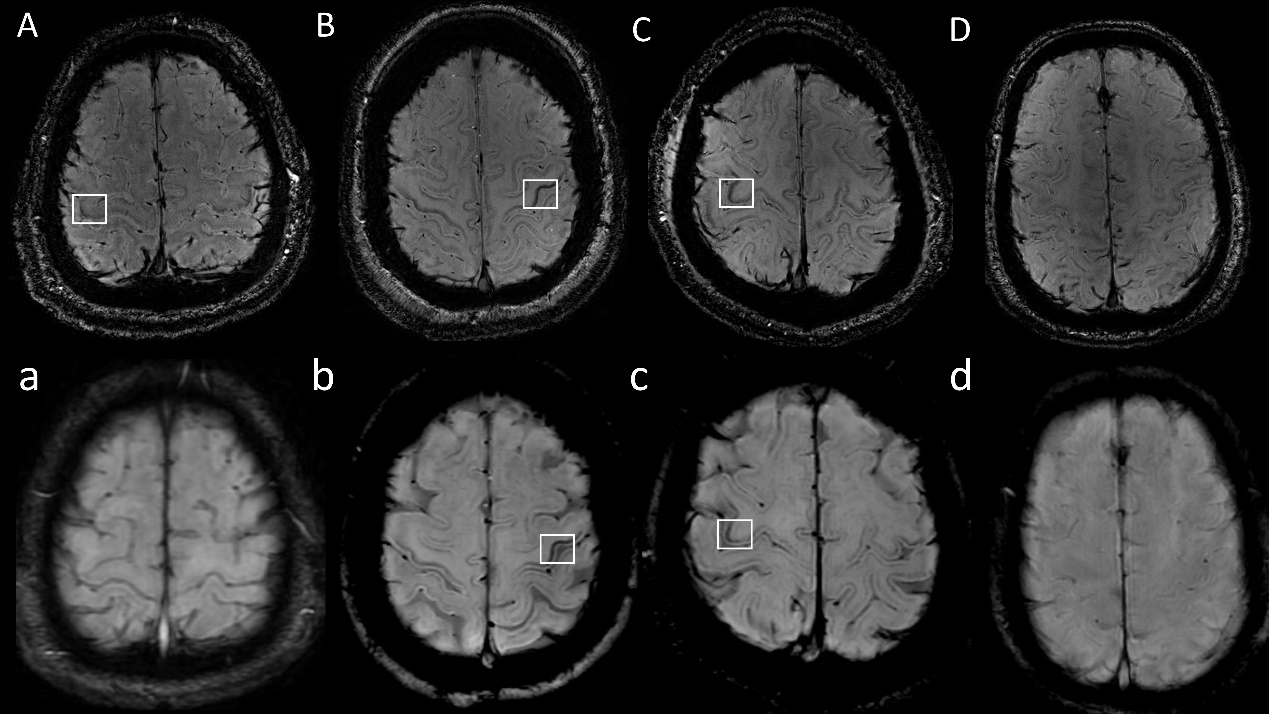


**Fig. S7 7T and 3T SWI images of other 4 ALS patients**

Upper panels A-D display 7T SWI images from the other four ALS patients, with corresponding 3T SWI images in lower panels a-d. White boxes indicate MBS confirmation using the MBHR threshold of 54.6%. The MBS was identified in three of the shown 7T cases and two of the 3T cases.


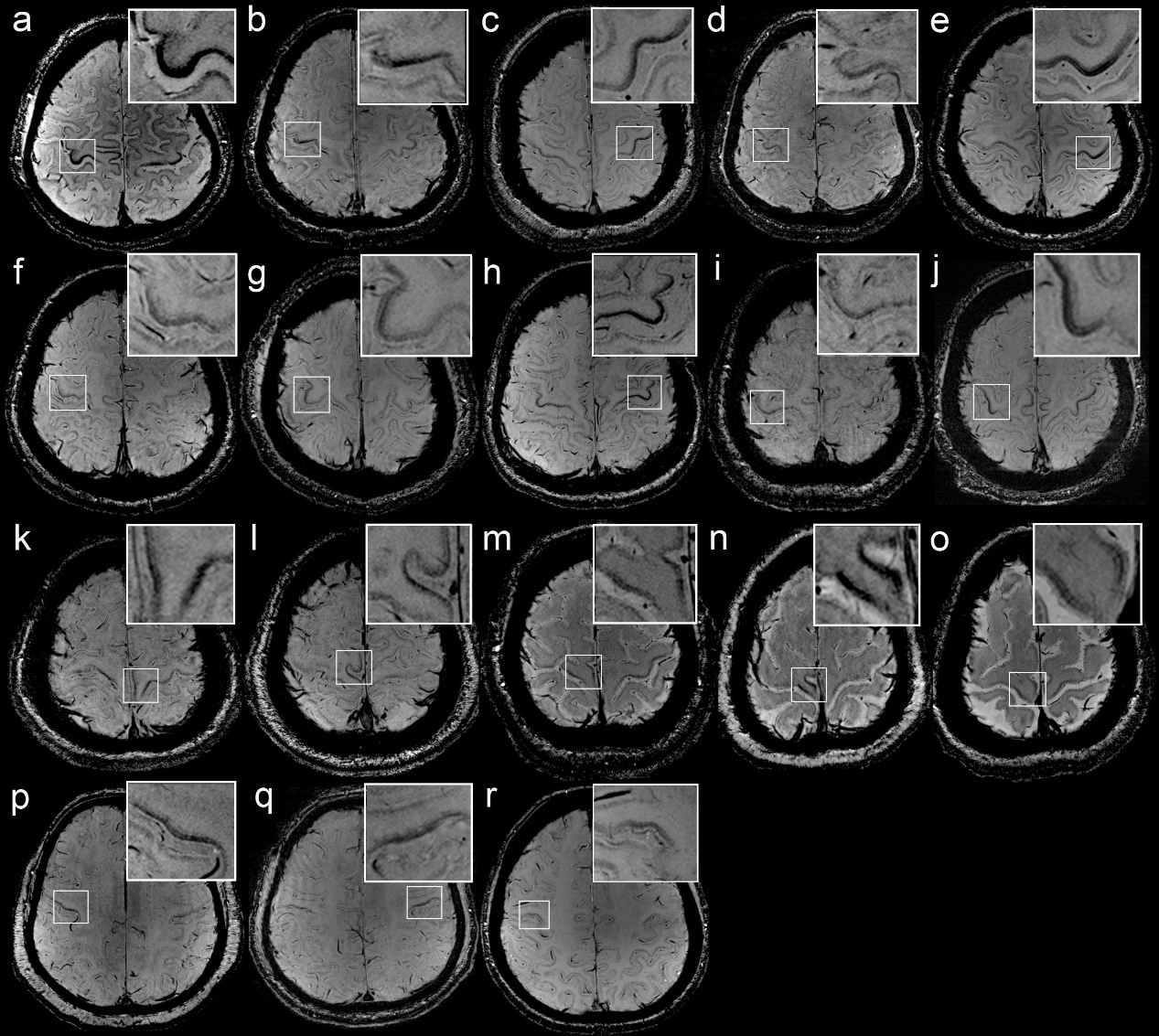


**Fig. S8** Motor band signs in 7T SWI of clinically definite ALS patients a-r.

**
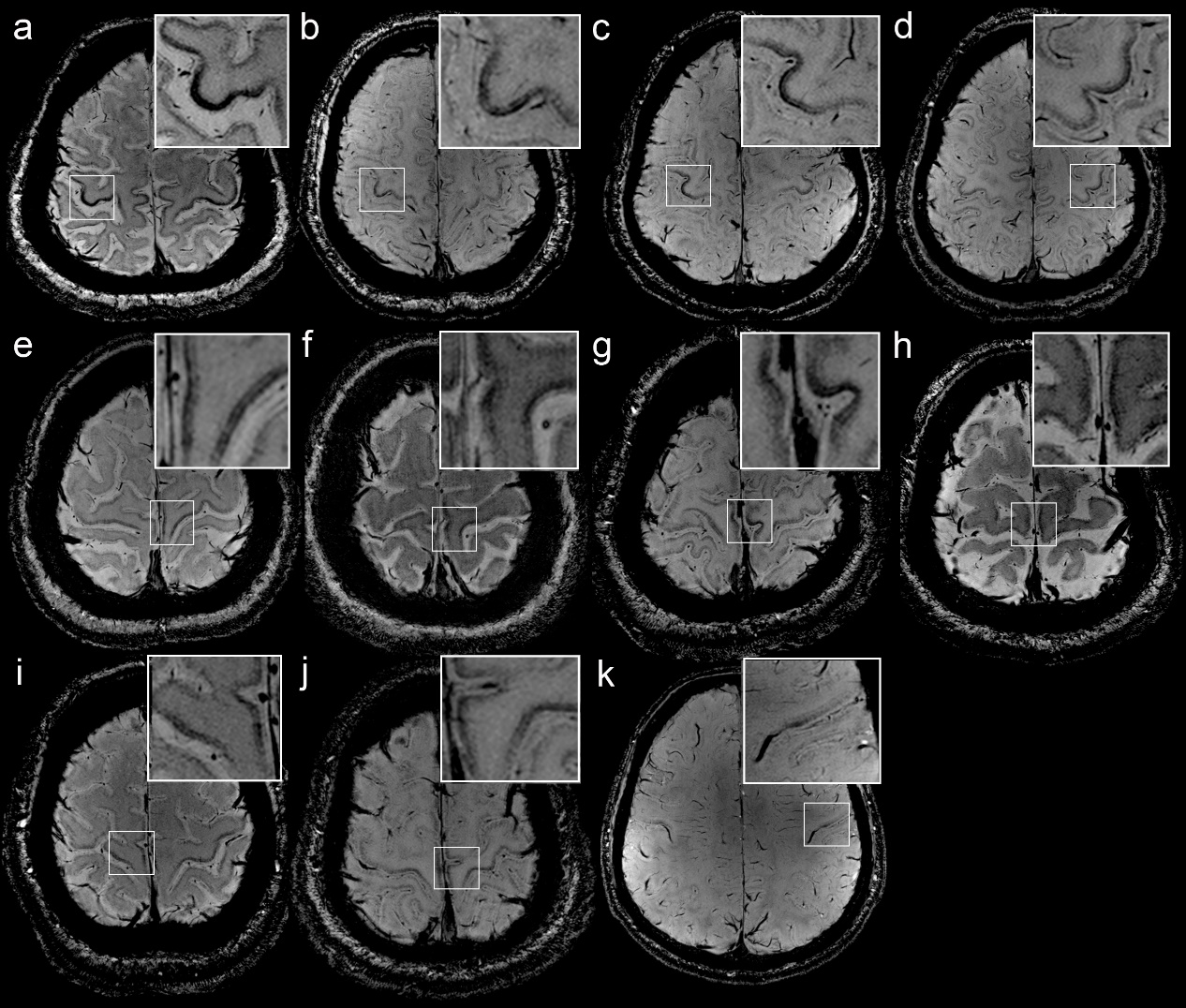
**

**Fig. S9** Motor band signs in 7T SWI of clinically probable ALS patients a-k.

**
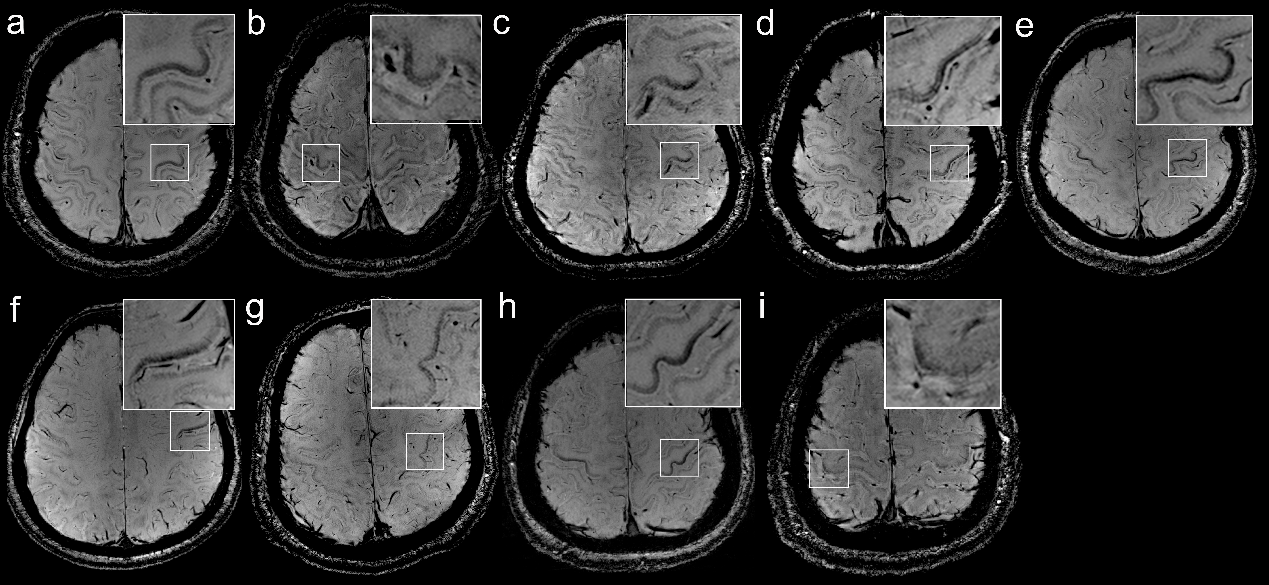
Fig. S10** Motor band signs in 7T SWI of clinically probable laboratory–supported ALS patients a-f; and clinically possible ALS patients g-i.
