# Supplementary material for: Diagnostic value of the motor band sign in amyotrophic lateral sclerosis: a 7T magnetic resonance imaging study
Source: Transl Neurodegener. 2025 Jun 18;14:30. doi: 10.1186/s40035-025-00491-8 (PMC12175456; doi:10.1186/s40035-025-00491-8)
Supplement: Supplementary file 2 — Additional file 2. Methods. [file 40035_2025_491_MOESM2_ESM.docx]

**Clinical assessment**

All subjects underwent imaging and electrophysiological examinations and had their clinical data collected within a week. The clinical data included duration, the MGH Upper Motor Neuron Scales (MGH UMNSs) [1], the Amyotrophic Lateral Sclerosis Functional Rating Scale-Revised (ALSFRS-R), etc. Two experienced neurologists (H. P., F. J.) conducted clinical examinations jointly and reached a consensus, especially for indeterminate cases. The clinical data of ALS mimics and healthy controls (HCs) were also collected simultaneously.

The MGH UMNS [1] (scored range from 0 to 45) was used to assess UMN impairment (containing jaw jerk, biceps reflex, triceps reflex, brachioradialis reflex, and Hoffmann’s sign, patellar tendon reflex, Achilles tendon reflex, Babinski reflex).

For longitudinal monitoring, ALS patients and mimics received structured telephone follow-up assessments at 6-month intervals (allowable window: ±2 weeks), and the outcomes were based on the last follow-up before enrollment expiration. Twelve ALS patients were excluded from the follow-up owing to personal reasons. The ALSFRS-R [2] (scores ranged from 0 to 48) was utilized to assess the degree of disability of ALS patients and ALS mimics at baseline (ALSFRS-R1) and follow-up (ALSFRS-R2). Their corresponding disease duration information was also collected at baseline (Duration 1) and follow-up (Duration 2). The disease progression rate (ΔFS) was calculated in months using the following formula [3-8]:

$$\text{ΔFS=}\frac{\text{ALSFRS-R1 - ALSFRS-R2}}{\text{Duration 2 - Duration 1}}$$

ALS patients demonstrating stable ALSFRS-R scores during follow-up will undergo repeat electromyography (EMG) and clinical reassessment, with subsequent exclusion of cases failing to meet diagnostic confirmation criteria.

**MRI parameters**

All participants underwent a 7T MRI (MAGNETOM Terra, Siemens Healthcare, Erlangen, Germany) scan using a 32-channel head coil (Nova Medical, Wilmington, Massachusetts, USA). The MRI protocol included whole-brain three-dimensional T1-weighted imaging (T1WI) and three-dimensional SWI. The parameters were as follows: T1WI (MP2RAGE): sagittal 3D scan, TE/TR/TI1/TI2=3.27/4500/1000/3200ms, slices=240, voxel size=0.8×0.8×0.8mm^3^; SWI: axial 3D scan, TE/TR=12/19ms, slices=104, voxel size=0.3×0.3×1.2mm^3^. Clinical SWI data were acquired using 3T clinical scanners from multiple manufacturers, including GE Healthcare (Chicago, IL, USA), Siemens Healthineers (Erlangen, Germany), and Philips Healthcare (Best, The Netherlands).

**The MBHR measurement protocol**

To date, no standardized evaluation protocol exists for MBS assessment. Visual interpretation of MBS remains susceptible to subjective interpretations, while quantitative approaches necessitate reference tissue selection for signal intensity normalization. Earlier investigations often use the cerebrospinal fluid (CSF), splenium of the corpus callosum (SCC), or other white matter components as reference regions of the MBS [4, 9-11]. However, they have their own limitations. Firstly, the CSF may not be an ideal reference due to variations in signal intensity caused by flow, field inhomogeneity, and surrounding structures like the choroid plexus [12, 13]. However, the patient-specific B1 transmit magnetic field inhomogeneity at 7 Tesla can induce substantial signal fluctuation across field of view (FOV) [14]. Different brain regions may suffer different levels of signal attenuation, especially when they are far from each other (e.g. motor cortex and CSF/SCC).

To address this confounding factor, we implemented a clinically simple and feasible standardized reference selection protocol in which subcortical white matter areas were strategically sampled within the MBS 5 mm range in the same axial sections. Although magnetic field inhomogeneity effects persist, they can be minimized during the ratio calculation (during the ratio calculation, the ROIs for both the numerator and the denominator are positioned very closely, which largely offsets any signal non-uniformity) compared to traditional methods using CSF and SCC reference, albeit empirically.

The following is the assessment course: two neuroradiologists blinded to clinical information manually outlined the ROIs using the MR workstation (syngo MR workspace, Siemens Healthcare, Erlangen, Germany) (Fig. 1A), and the motor band hypointensity ratio (MBHR) was applied to define the MBS: The SWI image was navigated to the precentral gyrus using the T1WI sequence as an anatomical reference. Axial slices demonstrating hypointense signals in the motor band sign (MBS) region were systematically identified through slice-by-slice visual inspection. The signal intensity measurement was performed using workstation's region-of-interest (ROI) analysis module, and the axial slices exhibiting the most pronounced signal attenuation were selected for motor band hypointensity ratio (MBHR) calculations.

To account for layer-specific iron deposition patterns and minimize potential noise interference, we implemented the cortical laminar analysis protocol described by Yu et al. [15]. Six circular ROIs were delineated on identical imaging planes:

1.**Pathology-targeted ROIs**: Three ROIs (ROI 1-3) positioned in the M1 areas exhibiting minimal signal intensity, identified through comparative visual analysis and quantitative measurements.

2.**Reference ROIs:** Three ROIs (ROI 4-6) within adjacent brightest subcortical white matter, maintaining 5 mm spatial separation from the grey-white matter interface.

The signal intensities in each ROI were recorded, and MBHR was calculated as the equation:


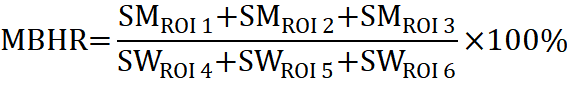


where SM_ROI 1, 2, and 3_ indicate the signal intensity of ROIs in M1, and SW_ROI 4, 5, and 6_ indicate the signal intensity of ROIs in adjacent subcortical white matter. Each ROI was drawn at the size of about 25 voxels.

This protocol was also implemented in the analysis of 3T SWI images.

**Statistical analysis**

SPSS Statistics software (version 26; IBM; Armonk, NY, USA) was used for statistical analysis in this study. The level of significance was fixed at *p* < 0.05. The Shapiro-Wilk test was used to determine the normality of continuous variables, and Levene's test was employed to determine variance equality. Categorical variables were indicated as cases (percentages); age, MGH UMNSs, MBHR were expressed as mean (standard deviation [SD]); and disease duration, ALSFRS-R, mean time between baseline and follow-up, and ΔFS were expressed as median (interquartile range [IQR]). The Chi-square test (gender) and Kruskal-Wallis H test (age, MGH UMNSs) (Post-hoc pairwise comparisons with Bonferroni correction were conducted following significant Kruskal-Wallis results, with adjusted p-values reported) were utilized to compare demographic data between ALS patients, ALS mimics, and HCs. Mann-Whitney U test was used to evaluate other clinical data (disease duration at baseline, interval between baseline and follow-up, ALSFRS-R, ΔFS) from ALS patients and ALS mimics. The MBHR between groups was examined using the Kruskal-Wallis H test. The statistical methods for group comparisons used in Tables S2 and S4 (Additional file 1) are annotated below each table. The Spearman’s test was applied to determine the correlation between MBHR and ΔFS. The Pearson’s test was applied to determine the correlation between MBHR and age, MGH UMNSs. The intraclass correlation coefficient (ICC) was used to compare the consistency between the two observers.

The performance of MBHR in distinguishing ALS subgroups from all controls (combining ALS mimics and HCs) was evaluated using the receiver operating characteristic (ROC) curve. The MBHR value with the Youden index (sensitivity- (1-specificity)) reaching its maximum was selected as the cutoff. The area under the curve (AUC), sensitivity, and specificity were also obtained.

**Intergroup consistency**

Two board-certified neuroradiologists independently performed blinded assessments using the above MBS evaluation scheme, with inter-rater reliability analysis revealing exceptional concordance (intraclass correlation coefficient [ICC] = 0.962, 95% confidence interval: 0.945-0.974; two-way random-effects model, absolute agreement definition). The statistically significant consistency (*p* < 0.0001) across 3 repeated evaluations at 2-week intervals underscores the reproducibility of this MBS quantification approach in high-field SWI applications.

**Reference**

1. Makary MM, A Weerasekara, H Rodham, et al. Comparison of Two Clinical Upper Motor Neuron Burden Rating Scales in ALS Using Quantitative Brain Imaging*.* ACS Chemical Neuroscience. 2021;12(5):906-916.

2. Cedarbaum JM, N Stambler, and E Malta The ALSFRS-R: a revised ALS functional rating scale that incorporates assessments of respiratory function. BDNF ALS Study Group (Phase III)*.* J Neurol Sci. 1999;169(1-2):13-21.

3. Cosottini M, G Donatelli, M Costagli, et al. High-Resolution 7T MR Imaging of the Motor Cortex in Amyotrophic Lateral Sclerosis*.* AJNR Am J Neuroradiol. 2016;37(3):455-461.

4. Bao Y, Y Chen, S Piao, et al. Iron quantitative analysis of motor combined with bulbar region in M1 cortex may improve diagnosis performance in ALS*.* Eur Radiol. 2023;33(2):1132-1142.

5. Walhout R, HJ Westeneng, E Verstraete, et al. Cortical thickness in ALS: towards a marker for upper motor neuron involvement*.* J Neurol Neurosurg Psychiatry. 2015;86(3):288-294.

6. van der Burgh HK, HJ Westeneng, R Walhout, et al. Multimodal longitudinal study of structural brain involvement in amyotrophic lateral sclerosis*.* Neurology. 2020;94(24):e2592-e2604.

7. Kimura F, C Fujimura, and S Ishida Progression rate of ALSFRS-R at time of diagnosis predicts survival time in ALS*.* Neurology. 2006;66(2):265-267.

8. Ferraro PM, F Agosta, N Riva, et al. Multimodal structural MRI in the diagnosis of motor neuron diseases*.* Neuroimage Clin. 2017;16:240-247.

9. Bhattarai A, GF Egan, P Talman, P Chua, and Z Chen Magnetic Resonance Iron Imaging in Amyotrophic Lateral Sclerosis*.* J Magn Reson Imaging. 2022;55(5):1283-1300.

10. Endo H, K Sekiguchi, H Shimada, et al. Low signal intensity in motor cortex on susceptibility-weighted MR imaging is correlated with clinical signs of amyotrophic lateral sclerosis: a pilot study*.* Journal of Neurology. 2018;265(3):552-561.

11. Costagli M, G Donatelli, L Biagi, et al. Magnetic susceptibility in the deep layers of the primary motor cortex in Amyotrophic Lateral Sclerosis*.* Neuroimage Clin. 2016;12:965-969.

12. LeVine SM, MJ Wulser, and SG Lynch Iron Quantification in Cerebrospinal Fluid*.* Anal Biochem. 1998;265(1):74-78.

13. Haacke EM, S Liu, S Buch, W Zheng, D Wu, and Y Ye Quantitative susceptibility mapping: current status and future directions*.* Magn Reson Imaging. 2015;33(1):1-25.

14. Williams SN, P McElhinney, and S Gunamony Ultra-high field MRI: parallel-transmit arrays and RF pulse design*.* Physics in Medicine & Biology. 2023;68(2):02TR02.

15. Yu J, F Qi, N Wang, et al. Increased iron level in motor cortex of amyotrophic lateral sclerosis patients: an in vivo MR study*.* Amyotroph Lateral Scler Frontotemporal Degener. 2014;15(5-6):357-361.
